# Precision Medicine in Systemic Mastocytosis

**DOI:** 10.3390/medicina57111135

**Published:** 2021-10-20

**Authors:** Maura Nicolosi, Andrea Patriarca, Annalisa Andorno, Abdurraouf Mokhtar Mahmoud, Alessandra Gennari, Renzo Boldorini, Gianluca Gaidano, Elena Crisà

**Affiliations:** 1 Division of Hematology, Department of Translation Medicine, Università del Piemonte Orientale and Azienda Ospedaliero-Universitaria Maggiore della Carità, Via Solaroli 17, 28100 Novara, Italy; andrea.patriarca@uniupo.it (A.P.); abdurraouf.mahmoud@uniupo.it (A.M.M.); gianluca.gaidano@uniupo.it (G.G.); elena.crisa@uniupo.it (E.C.); 2 Division of Pathology, Department of Health Sciences, Università del Piemonte Orientale and Azienda Ospedaliero-Universitaria Maggiore della Carità, 28100 Novara, Italy; annalisa.andorno@maggioreosp.novara.it (A.A.); renzo.boldorini@uniupo.it (R.B.); 3 Division of Oncology, Università del Piemonte Orientale and Azienda Ospedaliero-Universitaria Maggiore della Carità, 28100 Novara, Italy; alessandra.gennari@uniupo.it

**Keywords:** systemic mastocytosis, genetics, *KIT*, tyrosine kinase inhibitor, precision medicine

## Abstract

Mastocytosis is a rare hematological neoplasm characterized by the proliferation of abnormal clonal mast cells (MCs) in different cutaneous and extracutaneous organs. Its diagnosis is based on well-defined major and minor criteria, including the pathognomonic dense infiltrate of MCs detected in bone marrow (BM), elevated serum tryptase level, abnormal MCs CD25 expression, and the identification of *KIT* D816V mutation. The World Health Organization (WHO) classification subdivides mastocytosis into a cutaneous form (CM) and five systemic variants (SM), namely indolent/smoldering (ISM/SSM) and advanced SM (AdvSM) including aggressive SM (ASM), SM associated to hematological neoplasms (SM-AHN), and mast cell leukemia (MCL). More than 80% of patients with SM carry a somatic point mutation of *KIT* at codon 816, which may be targeted by kinase inhibitors. The presence of additional somatic mutations detected by next generation sequencing analysis may impact prognosis and drive treatment strategy, which ranges from symptomatic drugs in indolent forms to kinase-inhibitors active on *KIT*. Allogeneic stem cell transplant (SCT) may be considered in selected SM cases. Here, we review the clinical, diagnostic, and therapeutic issues of SM, with special emphasis on the translational implications of SM genetics for a precision medicine approach in clinical practice.

## 1. Introduction

Mastocytosis is a rare hematological neoplasm characterized by heterogenous clinical manifestations due to the excessive proliferation of abnormal clonal mast cells (MCs) in different cutaneous and extracutaneous sites, such as bone marrow (BM), spleen, lymph nodes, and the gastrointestinal (GI) tract [1]. Mastocytosis has historically been a diagnostic challenge due to the constellation of presenting symptoms, thus resulting in underestimation of disease incidence and prevalence. The estimated prevalence of systemic mastocytosis (SM) is 1/10,000 adults, and the estimated incidence amounts to 1/100,000 per year [2]. 

The clinical presentation of mastocytosis is very heterogeneous and ranges from skin-limited disease in cutaneous mastocytosis (CM), as typically observed in pediatric cases with a spontaneous regression in puberty, to diffuse extracutaneous involvement with more aggressive presentations in adult patients, named systemic mastocytosis (SM) with multiorgan dysfunction and shorter survival [3,4,5]. The clinical approach to SM requires multidisciplinary collaboration of hematologists, gastroenterologists, dermatologists, specialists of bone diseases, and allergogists [6]. The increased knowledge in SM genetics and the wider availability of molecular diagnostic tests have resulted in an improvement in the recognition and treatment of this rare disease. Approximately 90% of mastocytosis is associated with somatic gain-of-function point mutations in *KIT* [7]. However, the presence of additional somatic mutation in different genes, such as *SRSF2*, *ASXL1*, *RUNX1*, *EZH2,* and loss of function of *SETD2*, may impact the disease course, especially in the aggressive forms of SM, and may drive a personalized therapeutic approach [8,9,10]. Indeed, adult SM is managed with a tailored approach ranging from symptomatic treatment aimed at reducing the release of the MCs mediators and controlling their effects in indolent SM to target cytoreductive therapy in the aggressive variants of SM. 

Historically, SM has been treated with interferon-α (INF-alfa), cladribine, and imatinib in selected cases; however, the advent of kinase inhibitors targeting the *KIT* receptor offered encouraging perspectives [11,12]. Allogeneic stem cell transplantation (SCT) may be an option in selected SM associated with hematological neoplasms (SM-AHN) [13,14]. Due to heterogeneous presentation and the unpredictable behavior of the different variants of SM, correct classification and accurate risk assessments are mandatory in order to offer the best treatment options to SM patients. Here, we review the clinical, diagnostic, and therapeutic issues of SM, underlining how the recent impact of molecular biology may guide the approach of clinicians.

## 2. Clinical Presentation and Diagnostic Work-Up

According to the 2016 revision of World Health Organization (WHO) classification of myeloid neoplasm, mastocytosis is considered a distinct entity within myeloproliferative neoplasms (Table 1) [1]. Clinical presentation is widely heterogenous, and the multisystemic involvement may require a multidisciplinary diagnostic approach. Clinicians should conduct comprehensive evaluations of signs and symptoms with special consideration of triggers of MCs activation and a complete medical history, including the presence of repeated anaphylactic episodes after Hymenoptera sting. Unexplained osteoporosis, especially in men, as well as unexplained lymphadenopathy and/or splenomegaly, may trigger the suspicion of mastocytosis.

**Figure 1:**
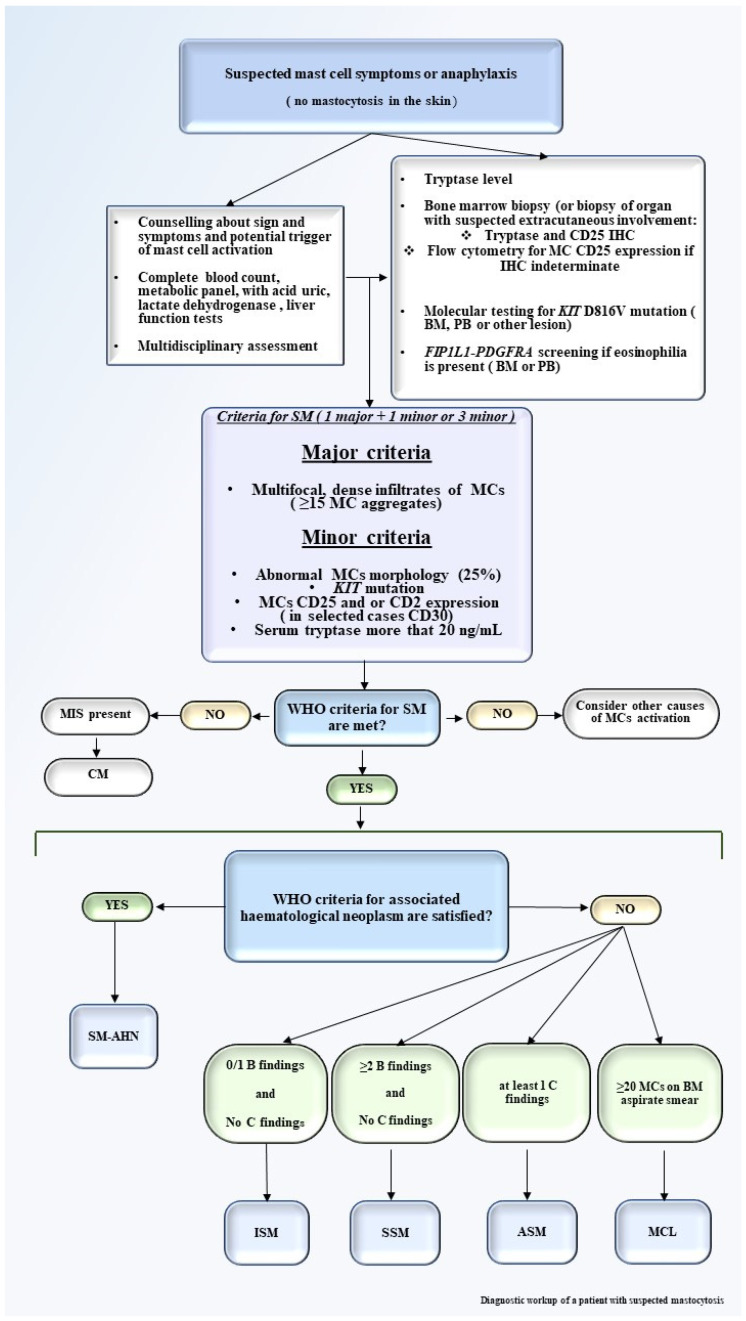
Diagnostic workup of a patient with suspected mastocytosis. The diagnostic workup of a patient with suspected mastocytosis should start with personal medication history, physical examination, counseling about triggers that may increase MCs activation, and collection of signs and symptoms. A multidisciplinary approach is useful. Laboratory tests include CBC with manual differential, serum tryptase level, BM biopsy with IHC for detection of CD25, and flow cytometry for MCs CD25 and in selected cases of CD30 expression if IHC is not available [21]. Molecular testing for *KIT* D816V mutation (in BM, PB, or other lesions) should be performed, as well as for *FIP1L1-PDGFRA* if eosinophilia is present. If available, the NGS test may be assessed. Major and minor criteria should be reached. After the identification of SM, B and C findings should be defined for the correct identification of SM variants. Abbreviations: MCs, mast cells; CBC, complete blood count; BM, bone marrow; IHC, immunohistochemistry; PB, peripheral blood; SM, systemic mastocytosis; NGS, next generation sequencing; MIS, mastocytosis in the skin; SM-AHN, systemic mastocytosis associated with a hematological neoplasm; ISM, indolent systemic mastocytosis; SSM, smoldering systemic mastocytosis; ASM, aggressive systemic mastocytosis; MCL, mast cell leukemia.

Laboratory tests may evidence eosinophilia, elevated acid uric and lactate dehydrogenase (LDH), alteration in liver function tests (LFTs), and increased serum tryptase level, which is an important indicator of MCs activation. A key point of mastocytosis diagnosis is represented by the bone marrow aspirate and bone marrow biopsy or biopsy of extracutaneous involved organ. Flow cytometry and immunohistochemistry are both mandatory for diagnosis [17]. On the contrary, molecular testing on peripheral blood (PB) or BM to detect *KIT* D816V or other less frequent *KIT* mutations, as detailed below, by conventional polymerase chain reaction (PCR) or allele-specific oligonucleotide quantitative PCR (ASO-qPCR) especially in low MC burden plays a crucial role in diagnostic work-up and also has therapeutic implications [18,19]. FISH is required in SM-AHN cases. NGS analysis with a myeloid mutation panel, as detailed below, may increase the information in terms of survival [18,20]. A proposed diagnostic framework for mastocytosis is proposed in Figure 1.

**Figure 2:**
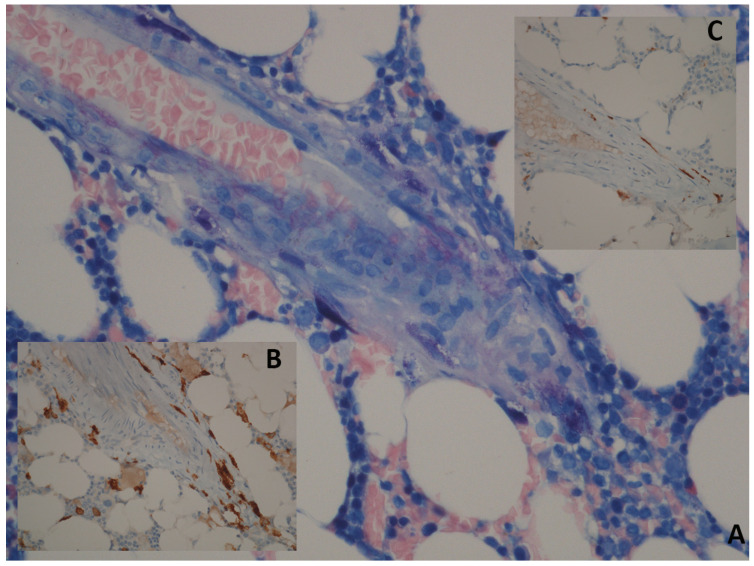
Bone marrow histology of patient affected by SM. Panel (**A**): Giemsa; 40×: perivascular spindle-shaped mast cells with abnormal cytologic features (spindling and hypogranularity). Panel (**B**): CD117; 40×: in tissue sections, an immunohistochemical stain can be used for identification of mastcells. Panel (**C**): immunostaining with CD25 shows an atypical immunophenotype of mast cells with membrane reactivity.

The WHO has proposed well-defined major and minor criteria for diagnosis (Table 2) [1]. The diagnosis of SM is established when major criteria and at least one of the minor criteria, or at least three minor criteria, are present. BM is almost always involved in adult mastocytosis, showing clusters of abnormal MCs for morphology (fusiform, with polar cytoplasmic process and cytoplasmatic hypo-granularity) and phenotypes (abnormal MCs usually express CD25). In addition, BM examination may allow the identification of an associated hematologic neoplasm (Figure 2) [22,23,24,25]. The abnormal MCs usually express CD25, which is considered a more representative marker than CD2 [17,26]. Elevated levels of serum tryptase are virtually detected in all SM cases but are not specific for mastocytosis, since they may also increase in different myeloid neoplasms, namely acute myeloid leukemia (AML), chronic myeloid leukemia (CML), and myelodysplastic syndromes (MDS). When the diagnostic criteria for SM are met, SM is then subclassified by the identification of additional “B” and “C” findings summarized in Table 3. Based on the aforementioned clinical, histological, immunophenotypic, and biological aspects, SM is classified into five variants: indolent systemic mastocytosis (ISM), smoldering systemic mastocytosis (SSM), systemic mastocytosis with an adjunctive hematological neoplasm (SM-AHN), aggressive systemic mastocytosis (ASM), and mast cell leukemia (MCL) [27]. ASM, SM-AHN, and MCL have been defined as “advanced SM” (AdvSM) due to their worse prognosis compared to the other subtypes [5,27]. A precise identification of the SM subtype is a relevant goal of the diagnostic framework of mastocytosis due to its implications for the therapeutic approach.

Indolent and Smoldering Systemic Mastocytosis. ISM is the most common variant of SM and is characterized by a slowly progressive clinical course with life expectancy comparable to the general population. The diagnosis of ISM meets the criteria of SM in the absence of the “B or C finding”. ISM has relatively good prognosis in terms of overall survival (OS) and leukemia-free survival (LFS) compared with ASM and SM-AHN [5,28]. SSM is a new SM category in the WHO classification, previously mentioned as a provisional subvariant of ISM. Compared to ISM, SSM was associated with older age, as expected from disease definition, with higher bone marrow MC burden, higher serum tryptase level, and higher prevalence of palpable hepatomegaly and splenomegaly. SSM is currently considered an intermediate variant and is characterized by the presence of ≥ 2 “B findings”, thus, a high burden of MCs [29]. SSM prognosis is better than AdvSM but worse than ISM [29,30,31]. 

Aggressive Mastocytosis. Aggressive SM is characterized by the presence of “C findings”. Patients may display constitutional symptoms, hepatosplenomegaly, lymphadenopathies, severe anemia and/or thrombocytopenia, leukocytosis, and very high serum tryptase levels. Due to the massive release of mediators by MCs, clinical manifestations may be present in different organs and systems, including the (GI) tract with abdominal pain, diarrhea, nausea vomiting, peptic ulcer, and GI bleeding [32,33,34]. Depression, musculoskeletal pain, and osteopenia with or without osteoporosis may also occur [34,35]. In a series of 342 SM patients, the OS of ASM was 41 months [5].

Systemic Mastocytosis with an Associated Hematological Neoplasm (SM-AHN). SM-AHN displays a more aggressive clinical course versus the other aforementioned variants and it is frequently observed in older patients [5]. Hematological malignancies mostly associated with SM include chronic myelomonocytic leukemia (CMML), MDS, myeloproliferative neoplasms (MPN), AML, B-cell lymphoma, and plasma cell neoplasms [36]. Among 342 consecutive patents with SM, 123 had an SM-AHN subdivided in SM-MPN (45%), SM-CMML (29%), SM-MDS (23%), and SM-AL (3%) [37]. After a median follow-up of 15 months, 90 (73%) deaths were recorded (100% with SM-AL, 89% SM-CMML, 82% SM-MDS, and 56% with SM-MPN). SM-MPN cases had significantly longer median survival (31 months) compared with SM-CMML, SM-MDS, and SM-AL [37]. Leukemic transformation is higher in SM-MDS than in SM-CMML [37].

Mast Cell Leukemia. MCL is a rare and extremely aggressive variant of SM and is characterized by the highest mortality. It is also considered a form of acute leukemia, defined by the presence of at least 20% neoplastic MCs in the BM and 10% in the PB. The spread of MCs may affect different organs with typical aggressive manifestations, such as constitutional symptoms, cytopenia, hepatic malfunction, hypersplenism, and malabsorption [38,39]. In addition to driver mutation *KIT* D816V, somatic mutations in *SRF2*, *ASXL1*, *RUNX1*, *TET2*, *CBL*, *K/N-RAS*, and *EZH2* have been identified in MCL patients, explaining, at least in part, the more aggressive course and poorer survival of MCL compared to other SM variant [40]. 

## 3. Molecular Aspects: *KIT* and beyond as Biomarkers of the Disease

MCs originate from hematopoietic progenitor cells in the BM and, after a partial differentiation, are released as precursors in the bloodstream; reach tissues and organs; and complete their maturation, differentiation, and proliferation [41]. These processes and the survival of MCs are strongly dependent upon the binding of stem cell factor (SFC) to the extracellular domain of the *KIT* receptor [42,43]. 

**Figure 3:**
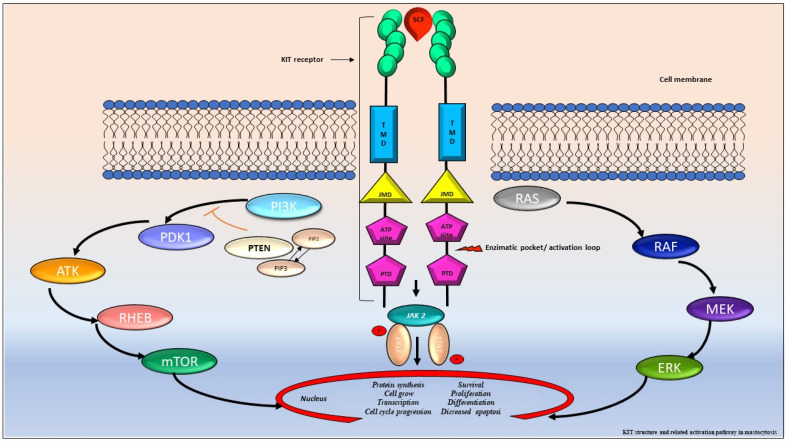
*KIT* structure and related activation pathway in mastocytosis. The proto-oncogene *KIT* encodes a type III tyrosine-kinase (TK) receptor, consisting of an extracellular domain (ECD) with five immunoglobulin-like motives, that includes the stem cell factor (SCF) binding site, a transmembrane domain (TMD), a juxta membrane domain (JMD), and two catalytic tyrosine kinase domains with ATP and phosphotransferase domain (PTD) binding site, separated by a kinase insert [42,44]. In normal conditions, the SCF ties the *KIT* binding site leading to receptor dimerization, autophosphorylation and kinase domain activation. Consequentially, it triggers a cascade of multimolecular phosphorylation resulting in different signal transduction pathways, such as the Janus kinase (JAK) and the signal transducers and activators of transcription (STAT), the phosphatidylinositol triphosphate kinase (PI3K), the rat sarcoma (RAS), and extracellular signal-regulated kinase (ERK) pathway [42,47,48,49]. In SM, *KIT* is constitutively activated, resulting in MCs proliferation, differentiation, survival, migration, and cytokine production.

The *KIT* proto-oncogene is located on the long arm of chromosome 4 (4q11–4q13) and contains 21 exons (Figure 3). *KIT* encodes a type III tyrosine-kinase (TK) receptor, which is detailed in Figure 3 [42,44]. In SM, *KIT* mutations result in the constitutive activation of the receptor, resulting in MCs proliferation, differentiation, survival, migration, and cytokine production. Indeed, more than 90% typical ISM and 70% of AdvSM carry an acquired point mutation in the *KIT* gene. The most common activating loop mutation of *KIT* is D816V, replacing aspartic acid with valine at codon 816 and resulting in its constitutive activation [45]. Occasionally, other variants targeting the same codon, such as D816Y, D816F, D816H, and D816I, have been described [46].

The advent of NGS allowed the deep molecular characterization of SM patients beyond the sole *KIT* mutation. Additional somatic mutations (e.g., *TET2*, *SRSF2*, *ASXL1*, *EZH2*, *CBL*, *RUNX1*, and *RAS*) have been found in 90% of AdvSM patients, especially in SM-AHN [50,51,52]. In a series of 70 patients affected by AdvSM, the most frequently mutated genes identified by NGS were *TET2* (47%), *SRSF2* (43%), *ASXL1* (29%), *RUNX1* (23%), *JAK2V617F* (16%), *N/KRAS* (14%), *CBL* (13%), and *EZH2* (10%). Less frequently mutated genes were *IDH2*, *ETV6*, *U2AF1*, *SF3B1*, *MLL*, *NPM1*, *DNMT3A*, and *TP53*. Sixty percent of patients harbor two or more mutations, in addition to *KIT* D816V. Mutations in *SRSF2* and *ASXL1* are independent predictors that adversely influence OS. A worse OS associates with the number of concurrent mutations in *SRSF2/ASXL1/RUNX1* (S/A/R) that have been incorporated in the mutation adjusted risk score (MARS) system for SM [52]. A similar panel of mutated genes has been described by Pardanani et al. in a study of 150 cases of SM and myeloid diseases. The most frequently mutated genes were *TET2* (29%), *ASXL1* (17%), and *CBL* (11%), with a significantly higher mutation frequency in SM-AHN. *ASXL1* and *RUNX1* mutations were associated with inferior survival in AdvSM [53]. Based on the impact of mutations on OS, molecular information in addition to *KIT* D816V identification has been incorporated into prognostication risk assessment.

Recently, great attention has been given to the *SETD2* gene due to its implication in cancer. The human *SETD2* gene is located at cytogenetic band p21.31 of chromosome 3, a region frequently targeted by copy number loss in various tumors [54]. The SETD2 protein consists of 2564 amino acids and has a molecular weight of 287.5 KD. SETD2 is responsible for tri-methylation of lysine 36 on histone H3 (H3K36me3) that is correlated with transcriptional activation and also contributes to DNA double-strand break repair in response to DNA damage [55,56]. Martinelli et al. reported loss of function mutations of *SETD2* in a series of 53 SM patients and have suggested that reduced SETD2 expression/absence and H3K36Me3 deficiency might potentiate the effects of *KIT* constitutive activation to determine the phenotype of advanced SM [54]. 

## 4. Cytogenetic Information

Cytogenetic analyses are performed at diagnosis in a minority of patients with AdvSM, and few data are available. Previous data reported similar cytogenetic abnormalities in SM and in MPN, such as trisomy 8, trisomy 9, and del(20)(q11), suggesting common pathogenetic pathways in MSc and myeloid disorders [57]. In a study of 34 patients with either urticaria pigmentosa or systemic mastocytosis, chromosome abnormalities were found in 41% of the patients in at least one examination [58]. In a retrospective study of 109 patients with ISM and AdvSM with or without AHN, an aberrant karyotype was identified in 22% of cases [59]. According to their aberrant karyotype, the patients were stratified in two different cytogenetic groups. Normal karyotypes and favorable karyotypes such as del(5q), trisomy 8, del(1q), and del(12p) were classified in the good risk group, while complex karyotypes defined as ≥3 abnormalities and monosomy 7 were classified in the poor-risk group (*n* = 10). The median OS of poor-risk karyotype patients was significantly shorter than good-risk/normal karyotype patients, and the karyotype was confirmed as an independent prognostic variable in AdvSM [51,52]. In another large retrospective study of the Mayo Clinic on 348 consecutive SM, the karyotype was abnormal in 15% cases, including 6% of ISM, 26% of SM-AHN, 8% of ASM, and 28% of SM-AHN-myeloid (*p* < 0.001). No significant associations between abnormal karyotype and presence of adverse mutations have been demonstrated [60]. In univariate analysis, abnormal karyotype was associated with inferior survival in ASM and SM-AHN, but this finding was not confirmed in multivariate analysis. In this study, patients were also screened for the most common somatic mutations of myeloid disease, and the mutation status was prognostically more relevant than karyotype. In conclusion, no SM-specific cytogenetic aberrations have been identified, and information about the incidence and impact of cytogenetic aberrations in terms of survival is limited.

## 5. Risk Stratification

The five subgroups of SM identified by the WHO 2016 classification carry relevant differences in disease presentation and natural history of disease [1]. However, in addition to the WHO subtype, advanced age, history of weight loss, anemia, thrombocytopenia, hypoalbuminemia, and presence of BM blasts > 5% impact survival [5]. The advent of NGS has allowed greater accuracy in terms of prognostic stratification. Different studies have suggested different prognostic score systems combining clinical and molecular variables, summarized in Table 4. Pardanani et al. sequenced 27 genes in 150 SM patients and identified different mutations and variables that integrated into a clinical-molecular prognostic model allowing deep prognostication of AdvSM patients [53]. Based on these grounds, the Mayo Clinic group proposed the Mutation-Augmented Prognostic Scoring System (MAPSS), stratifying AdvSM into three distinct risk groups: low-risk (score 0–1.5), intermediate-risk (score 2–4.5), and high-risk (score 5–7.5) with median survival of 5, 21, and 86 months, respectively [61]. Jawhar et al. performed the MARS system for patients with AdvSM that integrates clinical and mutation characteristics. The study included 383 patients with AdvSM from the German Registry on Disorders of Eosinophils and Mast Cells as training set and a series from the European Competence Network on Mastocytosis as the validation set [62]. In multivariable analysis, age > 60 years, hemoglobin less than 10 g/dL, thrombocytopenia (platelets < 100 × 10^9^/L), presence of one high molecular risk gene mutation (i.e., *SRSF2*, *ASXL1*, and/or *RUNX1)* (S/A/R), and presence of two or more high molecular risk gene mutations were associated with shorter OS. The presence and number of gene mutations in the S/A/R panel had a strong adverse impact on OS. Three risk categories were defined: low risk (median OS, not reached), intermediate risk (median OS, 3.9 years), and high risk (median OS, 1.9 years). The MARS system was independent of the WHO classification type and was confirmed in the independent validation set [63]. 

Subsequently, the Mayo Alliance Prognostic System (MAPS) was developed; the clinical, cytogenetic, and molecular information of 580 patients, referred to Mayo Clinic from 1968 to 2015, was analyzed [63]. Two complementary risk models were elaborated: a clinical and a hybrid clinical-molecular model. The clinical model confirmed the independent prognostic contribution of five variables: WHO defined AdvSM type, platelets < 150 × 10^9^/L, increased ALP, age more than 60 years, and anemia. The hybrid clinical-molecular model replaced anemia with adverse mutations (i.e., *ASXL1*, *RUNX1*, and *NRAS*) as a risk factor [63]. Recently, an international study of the registry of the European Competence Network on Mastocytosis developed the International Prognostic Scoring System of Mastocytosis (IPSM) in a series of 1639 patients with SM [65]. IPSM divided patients with non-AdvSM into three groups based on age > 60 years and elevated ALP value: low (no risk factors), intermediate 1 (one risk factor), and intermediate 2 (two risk factors). In patients with AdvSM, age 60 years or older, a concentration of tryptase ≥ 125 ng/mL, a leukocyte count ≥ 16 × 10^9^/L, hemoglobin ≤ 11 g/dL or a platelet count ≤ 100 × 10^9^/L, and skin involvement were prognostic variables. Four risk categories were established for AdvSM with significantly different outcomes for OS and progression-free survival. IPSM was confirmed using a validation cohort from the Spanish network Red Española de Mastocitosis. IPSM is now commonly used to predict survival outcomes and to guide treatment decisions.

## 6. Treatment

**Figure 4:**
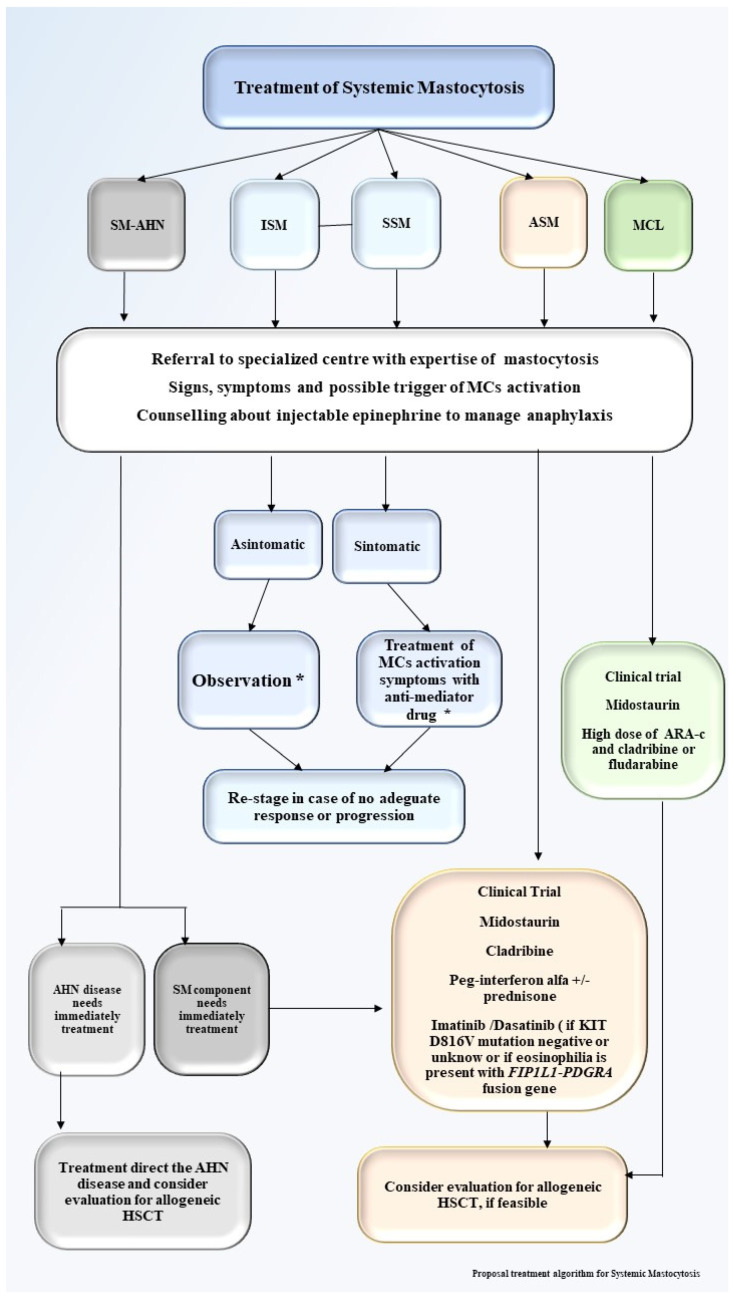
Proposal treatment algorithm for systemic mastocytosis. Proposal treatment algorithm for systemic mastocytosis. SM-AHN, systemic mastocytosis associated a hematological neoplasm; ISM, indolent systemic mastocytosis; SSM, smoldering systemic mastocytosis; ASM, aggressive systemic mastocytosis; MCL, mast cell leukemia; HSCT, hematopoietic stem cell transplant; * Enroll patients in clinical trial, if available.

Treatment of mastocytosis aims to control the symptoms of MCs activation and degranulation in indolent forms and to decrease MCs infiltration and reduce organ damage in the advanced forms. Major advances have occurred in the last 5 years regarding treatment of SM including new TKIs targeting *KIT*. Cytoreductive and immunomodulant drugs, such as cladribine or interferon, may be other options for disease control, and head-to-head comparisons with targeted therapies are lacking. A critical issue is whether the patient is eligible for allogeneic SCT and what might be the appropriate timing for transplant. Figure 4 summarizes the current available treatment options for SM.

Inhibition of MC activation and degranulation. Symptoms of SM can be managed by blocking the mediator receptors (H1 and H2 antihistamines; leukotriene receptor blockade), inhibiting mediator synthesis (aspirin and zileuton) or release (sodium cromolyn), by anti-IgE therapy, or by a combination of these approaches [66]. Combinations of baseline anti-H1 no-sedating medicines (e.g., cetirizine or levocetirizine) with the addition of sedating medications for breakthrough symptoms or at bedtime (such as diphenhydramine or hydroxyzine) and an H2 blocker are recommended [66]. H2 and proton pump inhibitors, such as ranitidine, cimetidine, or famotidine, can also address GI symptoms [66].

Patients with SM often suffer from MCs’ activation syndrome, which is mediated by clonal MCs, but in most cases the syndrome is also IgE-dependent with a high-risk of developing severe anaphylactic reactions [67]. Acute episodes of MCs activation require epinephrine, whereas prolonged episodes may be addressed with corticosteroids. Omalizumab, an IgE depleting drug, appears to prevent some life-threatening reactions associated with mastocytosis and may be a good option for treating the associated symptoms, although its use is supported only by observational and uncontrolled studies including small numbers of patients [68,69]. Neuropsychiatric symptoms associated with the presence of elevated MCs mediators, including anxiety or depression, may benefit of antidepressants and anxiolytic medications [70]. Purely symptomatic treatment may not suffice in some patients who require a reduction in MCs burden in order to prevent severe symptoms including anaphylaxis and/or progression to aggressive diseases.

Sarilumab. A phase 2 study to evaluate the safety and efficacy of Sarilumab in improving the quality of life in ISM is ongoing (NCT03770273). Sarilumab is approved by the Food and Drug Administration (FDA) for the treatment of rheumatoid arthritis [71]. The binding of sarilumab to the IL-6 receptor inhibits IL-6-associated human mast cell signaling and proliferation with decreased mediator release. Based on these grounds, sarilumab may be a rational choice for the treatment of ISM.

Siglec-8 targeting (AK002). In bone marrow aspirates from patients with SM, all activated mast cells display a robust expression of the Siglec-8 receptor [72]. A novel humanized monoclonal antibody to Siglec-8 (AK002) demonstrated SM mast cell inhibition in ex vivo bone marrow aspirates. AK002 also had depleting effects on eosinophils, which may be valuable to SM patients with associated eosinophilia. These encouraging results may represent a novel approach for the treatment of SM [72].

*KIT* inhibitors. The main advances in SM treatment are related to the inhibition of *KIT* D816V, which is the primary driver of MCs differentiation, proliferation, and survival in the overwhelming majority of patients.

Imatinib. *KIT* D816V is resistant to imatinib due to a conformational change in the enzymatic pocket that blocks the binding of the drug to the receptor, and nilotinib and dasatinib also lack significant clinical activity [73,74]. However, imatinib inhibits the growth of MCs with wild-type *KIT* or with mutations outside the *KIT* activation loop that target the extracellular (e.g., deletion of codon 419 on exon 8 or p.A502_Y503dup in exon 9), transmembrane (e.g., F522C), or juxtamembrane (e.g., V560G) domains. These mutations occur in <1% of all AdvSM cases and are enriched in cases of well-differentiated SM [75]. Imatinib has been approved by the Food and Drug Administration for SM patients negative for *KIT* D816V or with unknown *KIT* mutation status. A recent clinical trial and a systematic review carried out by the Red Española de Mastocitosis clarified that predictors of imatinib response in SM are the presence of imatinib-sensitive mutations involving *KIT* (e.g., juxtamembrane or transmembrane *KIT* mutations). Conversely, the sole absence of *KIT* D816V does not per se represent a positive predictor of imatinib response [76].

Midostaurin. Midostaurin is an inhibitor of PKC that can also target several clinically relevant kinases, including *KIT*, FLT3, PDGFRA, PDGFRB, vascular endothelial growth factor receptor KDR, and FES, both in their wild type and mutated forms [77]. In vitro, midostaurin was proven not only to induce apoptosis and growth arrest in *KIT* mutated cell lines and in neoplastic MCs from patients with AdvSM but also to suppress IgE-receptor mediated activation and mediator release in human MCs and blood basophils [78,79]. In a phase II trial of midostaurin in AdvSM on 89 patients with ASM, SM-AHN, or MCL, the overall response rate (ORR) was 60%, the median OS was 28.7 months, and the progression-free survival was 14.1 months [11]. Importantly, a decrease in mastocytosis related symptoms was observed in responding patients. At the dosage of 100 mg twice daily in 4-week cycles, the most common side-effects were low-grade nausea, vomiting, and diarrhea, whereas severe cytopenia occurred in <50% of patients. Of note, most patients were already cytopenic before treatment [11]. In another phase II trial on 26 patients with AdvSM and organ damage treated with the same midostaurin schedule, the median OS was 40 months (18.5 months for MCL patients) and the ORR was 69% at the median follow-up of 10 years, with clinical benefit in all AdvSM variants [12]. A single case report and a small patients series confirmed these results [80,81]. In patients with slow progression, treatment with midostaurin can induce major clinical responses with improvement or disappearance of C-findings, a decrease in MC burden in the BM and other organs, and a reduction in mediator-related symptoms in a significative number of patients, with moderate gastroenteric side effects [11,12,82,83,84,85,86,87]. Midostaurin may be used both as first-line treatment, especially in MCL patients, as well as salvage therapy in patients progressing after interferon-α, cladribine, or other cytoreductive therapy. Other possible roles for midostaurin include maintenance therapy after allogeneic SCT or for control of severe MCs activation symptoms [18].

Avapritinib (BLU-285). Avapritinib is an oral potent and selective tyrosine kinase inhibitor of PDGFRA and of the activation-loop mutants of *KIT*, including *KIT* D816V [88]. It is highly selective with limited inhibitory activity outside of *KIT* and PDGFRA kinases and has shown therapeutic activity in murine models of mastocytosis. A phase 1 trial (Explorer; NCT02561988) enrolled 52 AdvSM. Rapid antineoplastic activity was assessed by BM MCs burden, serum tryptase, and *KIT* D816V mutant allele burden. Among the 52 enrolled patients, only twenty-three were evaluable for response by IWG-MRT-ECNM criteria [89]. The ORR and the rate of CR plus CR with partial recovery of peripheral blood counts (CR/CRh) were 83% and 17%, respectively. Responses were obtained also in patients who experienced intolerance or no response relative to prior midostaurin. Adverse effects (AEs) were myelosuppression with thrombocytopenia and anemia, periorbital and peripheral edema, fatigue, nausea, vomiting, diarrhea, and cognitive effects. Treatment discontinuation occurred because of progession, AEs or inadequate response in 33%, 33%, and 17%, respectively. The protocol was modified to manage severe thrombocytopenia with strict dose interruption or reduction. Follow up of the phase 2 study (Pathfinder; NCT03580655) is ongoing. More data are needed to assess long-term responses and adverse effects of this novel TKI.

Ripretinib (DCC-2618). DCC-2618 is a new Type II switch pocket control inhibitor that has shown a potent inhibitory effect on exon 17 *KIT* mutations and that is resistant to other TKI [90]. The safety and tolerability of DCC-2618 in patients with advanced malignancies, including SM, is under study (NCT02571036). At the time of writing, the drug is approved for GastroIntestinal Stromal Tumors (GIST) by the FDA and European Medicines Agency (EMA).

Masitinib. In a phase 3 study, 135 patients with ISM or SSM were randomly assigned masitinib (*n* = 71) or a placebo (*n* = 64). By 24 weeks, masitinib was associated with a cumulative response of 18.7% in the primary endpoint (≥75% improvement from baseline within weeks 8–24 in at least one severe baseline symptom) compared with 7.4% for the placebo (*p* = 0.0076). Frequent severe adverse events included diarrhea (11% vs. 2% in the masitinib group vs. the placebo group), rash (6% vs. none), and asthenia (6% vs. 2%). The most frequent serious adverse events included diarrhea (4%) and urticaria (3%), and no life-threatening toxicities occurred. These findings indicate that masitinib may be an effective and well tolerated agent for the treatment of severely symptomatic ISM or SSM [91]. A phase 3 study comparing oral masitinib to placebo for the treatment of SSM or ISSM, unresponsive to optimal symptomatic treatment, is ongoing (NCT04333108).

BLU-263 (*KIT* inhibitor). BLU-263 is an investigational, potent, and selective oral small-molecule inhibitor of *KIT* with sub-nanomolar potency on D816V-mutant *KIT*. A randomized, double-blind, and placebo-controlled phase 2/3 study comparing the efficacy and safety of BLU-263 + best supportive care (BSC) with placebo + BSC in patients with ISM whose symptoms are not adequately controlled by BSC is currently recruiting (NCT04910685).

Bezuclastinib (PLX9486) PLX9486 has selective activity against primary *KIT* mutations (exons 9 and 11) and activation loop mutations (exons 17 and 18). Based on the results of PLX9486 in GIST, a recent interest has focused on the use of this drug on SM [92]. A phase 2 open-label multicenter clinical study of the safety, efficacy, pharmacokinetic, and pharmacodynamic profiles of CGT9486 (formally named PLX9486) as a single agent in patients with AdSM is ongoing (NCT04996875).

### 6.1. BLC-2 Inhibitors

**Obatoclax**. Recent data suggest that MCs in AdvSM express several antiapoptotic members of the Bcl-2 family, including Bcl-2, and myeloid leukemia cell differentiation protein 1 (Mcl-1) [93,94]. Obatoclax (GX015-070) is a novel BH3 mimetic small molecule type targeted drug that binds to and blocks the antiapoptotic activity of Mcl-1 and Bcl-2 [35,36,37,95]. Based on its activity in preclinical models, obatoclax has been recently investigated by Peter at al., showing that obatoclax may be an effective drug capable of suppressing the growth and survival in neoplastic MC in advanced SM [96]. Further studies are required to define its potential value and in vivo efficacy in advanced SM.

**Cladribine**. In two large series of SM patients treated with cladribine as single agent, the ORRs were 50% to 82% in patients with AdvSM and 60% to 92% in patients with ISM, with significant improvement in multiple symptoms [82,83]. In the study with the longer follow-up (>10 years), the median duration of response was 3.71 and 2.47 years for indolent and aggressive M, respectively, with immunosuppression and opportunistic infections as main toxicities and the latter occurring as grade 3 or 4 in a minority of patients (13%) [82]. Cladribine is active in all SM subtypes, with response rates similar to those reported with midostaurin, although there is no direct comparison, and the analysis is limited by small patient numbers. Cladribine may be used as first-line treatment when rapid disease debulking is needed or as salvage treatment in patients failing treatments [18]. 

**Interferon-α**. As reported since 1992, the administration of interferon alpha-2b has potential benefits in the treatment of mastocytosis [97]. INF-α and INF-α2b can reduce MCs degranulation and BM infiltration by MCs, can improve the typical cutaneous and GI signs, and can increase bone density [98,99,100]. INF-α is effective on all types of SM; however, the ORR is approximately around 20%, and no fixed dose and duration have been firmly established. Side effects, including cytopenia, liver toxicity and elevated transaminases, fatigue, nausea, fever, flu-like syndrome, and psychiatric sequelae, occur frequently, resulting in treatment discontinuation. To ameliorate tolerability and reduce discontinuation due to the side effects, INF-α has been administered in combination with prednisone [101]. In a series of 47 SM patients, INF-α with or without prednisone was administered. The dosage of INF-α ranged from 0.5 million units (MU) per day to 10 MU three times a week, and prednisone ranged from 20 mg to 60 mg per day. The ORR of the 40 evaluable cases was 53%, while CR, major response (MR), and PR were obtained in 3%, 15%, and 35% of cases, respectively [83]. The overall median duration of response was 12 months, and the responses were not significantly different between the arms with or without prednisone. 

**Allogenic stem cell transplant**. In a large cooperative study on 57 patients with SM who underwent allogeneic SCT, 40 patients (70%) achieved a response at day + 100. Sexteen patients (28%) achieved CR, including 2 patients who became *KIT* D816V-negative [13]. All 38 SM-AHN patients achieved a response, whereas half of the MCL patients were primary refractory. OS at 3 years was 57% for all patients, 74% in AHN group, 43% in AdvSM, and 17% in MCL group. Survival was lower in patients receiving reduced intensity conditioning compared with myeloablative conditioning and in patients in progression [13]. Allogeneic SCT can be considered in patients with associated hematological disease such as AML or in those with relapsed/refractory AdvSM [14,18]. In the event of a possible transplantation, a careful evaluation of the induction regiment is mandatory, preferring cladribine or small molecules to IFN based treatment due the increased risk of acute graft versus host disease (aGVHD) [14].

### 6.2. Future Perspectives

Despite many advances in the molecular genetics and precision medicine approach of SM, several issues deserve to be explored further with respect to this disease. From a therapeutic standpoint, CD123 is the α-subunit of the interleukin-3 receptor (ILR3) and represents a potential attractive therapeutic target in systemic mastocytosis (SM) given its absent expression on normal/reactive mast cells (MCs) and aberrant expression on neoplastic MCs. A recent study has suggested that targeting CD123 in SM may have direct and indirect anti-tumor effects [99]. A phase I study is currently investigating the best dose and side effects of the anti-CD123 flotetuzumab monoclonal antibody for the treatment of patients with CD123-positive relapsed or refractory hematological disease, including SM patients (NCT04681105) [100]. Tagraxofusp (SL-401) is a CD123-directed cytotoxin consisting of human interleukin-3 fused to truncated diphtheria toxin. It has been investigated in the blastic plasmacytoid dendritic-cell neoplasm (BPDCN), showing promising results [101], and deserves to be explored also in SM. Given a certain degree of heterogeneity in CD123 expression in SM, clinical trials based on anti-CD123-targeted therapy should be coupled with investigations aimed at correlating expression levels and outcome. As detailed above, the advances in understanding of biology and treatment of SM have been limited by the rarity of the disease and by the relative low number of MCs infiltrating the patients’ tissues. To overcome this limitation, Toledo et al. generated the induced pluripotent stem cells (iPSCs) from patients with aggressive SM, which represents a new approach for disease modeling and screening for precision medicine. Using these iPSCs, the authors identified nintedanib, which is a FDA approved angiokinase inhibitor that targets the vascular endothelial growth factor receptor (EGFR) and fibroblast growth factor receptor, as a novel *KIT* D816V inhibitor [102,103]. Table 5 summarizes the main recruiting clinical trials in SM.

The current scenario of rapidly changing paradigm for hematological malignancies mandates a systematic collection of patient-reported outcomes (PRO) and quality of life (QoL) data in both clinical research and in routine care [104]. Quality of life (QoL) and patient reported outcomes (PROs) have been explored to a limited extent in SM and deserve to be investigated in detail given the high burden of symptoms of many of these patients. A recent report has documented a high level of suffering and strong associations between impairments and symptom-related factors in the disease, pointing to the need of further addressing this issue [105]. Similarly to other myeloid malignancies, the use of disease adapted scoring systems for QoL and PROs may result in a better understanding of the patient’s perspective of symptoms and result in an improvement of disease management also in SM [106,107,108].

## 7. Conclusions

The diagnosis of SM might be challenging, and a multidisciplinary approach is desirable due to a broad variety of signs and symptoms driven by MCs degranulation. The disease pathogenesis is clearly driven by *KIT* mutations; however, new molecular technologies have allowed highlighting new genetic features that are involved in disease pathogenesis and may contribute to refining diagnostication and prognostication. In particular, the use of NGS has allowed building different prognostic scores, which may guide clinicians in terms of patient stratification and treatment choice. Resistance to the first generation TKI imatinib has resulted in exploring the potential utility of new selective TKIs, such as midostaurin and avapritinib, which have demonstrated activity against SM harboring the *KIT* D816V mutation. Encouraging results of anti-CD123 antibodies in hematological malignances may be investigated in MCs disease. Large collaborative efforts are needed to clarify the different open questions related to the diagnosis and treatment of SM.

## Figures and Tables

**Table 1:** WHO 2016 classification of mastocytosis.

Cutaneous Mastocytosis (CM)
Macupapular CM Diffuse CM Mastocytoma of skin
**Systemic Mastocytosis (SM)**
Indolent systemic mastocytosis (ISM) * Smoldering systemic mastocytosis (SSM) * Systemic mastocytosis with an associated hematological neoplasm (SM-AHN) Aggressive systemic mastocytosis (ASM) * Mast cell leukemia (MCL)
**Mast Cell Sarcoma (MCS)**

* Requires additional information regarding B and C finding for accurate diagnosis [15,16]. See Table 3 for details *.

**Table 2:** Diagnostic Criteria for Systemic Mastocytosis (SM) according to the 2016 WHO classification.

**Major criterion**	Multifocal, dense infiltrate of atypical MCs (≥15 MCs in aggregates) in BM biopsy and/or in section of other extracutaneous organs
**Minor criteria**	>25% of atypical or spindle shaped MCs in BM sections or other extracutaneous organs *KIT* point mutation at codon 816 in BM or PB or other extracutaneous organs MCs in BM or PB or other extracutaneous organs exhibit CD25 with or without CD2, in flow cytometry and immunochemistry, if available. Baseline serum tryptase value > 20 ng/mL

Abbreviations: WHO, World Health Organization; SM, systemic mastocytosis; MCs, mast cells; BM, bone marrow; PB, peripheral blood.

**Table 3:** Definition of B and C findings for Systemic Mastocytosis (SM).

**B—Findings**
Hight MCs infiltration in BM: ≥30% in histology and basal serum tryptase level > 200 ng/mL
BM with sign of dysplasia or myeloproliferation, without substantial cytopenia and without criteria of an associated hematological neoplasm and without sub
Organomegaly without impaired organ function: hepatomegaly, palpable splenomegaly, and/or palpable lymphadenopathy (or on CT or US)
**C—Findings**
BM dysfunction: ≥1 cytopenia (ANC < 1. 10^9^/L, Hbg < 10 g/dL, platelet < 100 × 10^9^/L)
Hepatomegaly and liver disfunction with ascites
Palpable splenomegaly with hypersplenism
Skeletal involvement with large size osteolysis with or without pathological fractures *
Malabsorption with hypoalbuminemia and weight loss
Live threatening organ damage in other organ systems due to local MCs infiltration

Abbreviations: MCs, mast cells; BM, bone marrow; CT, computerized tomography; US, ultrasound; ANC, absolute neutrophil count. * Pathological fractures caused by osteoporosis are not C-findings.

**Table 4:** Different prognostic score risk stratifications for Systemic Mastocytosis (SM).

MARS [64]		MAPS [63]		IPSS for No AdvSM [65]		IPSS for AdvSM	
Prognostic Variable and Points							
Age > 60 years	1	Age > 60 years	1	Age > 60 years	1	Age > 60 years	1
Hemoglobin < 10 g/dL	1	Advanced SM vs. ISM/SSM	2	ALP ≥ 100 U/L	1	Tryptase ≥ 125 ng/mL	1
Platelets < 100 × 10^9^/L	1	Platelets < 150 × 10^9^/L	1			Leukocytes ≥ 16 × 10^9^/L	1
One S/A/R (*SFRS2*, *ASXL*, or *RUNX1*) mutation	1	Serum ALP > normal range	1			Hemoglobin ≤ 11 g/dL	1
≥2 S/A/R mutation	2	Adverse mutation (*ASXL1*, *RUNX1* and *NRAS*)	1			Platelets <150 × 10^9^/L	1
						Skin involvement	−1
**Risk Group and Points**							
Low	0–1	Low	≤2	Low	0	AdvSM-1	−1 to 0
Intermediate	2	Intermediate- 1	3	Intermediate-1	1	AdvSM-2	1
High	3–5	Intermediate-2	4	Intermediate-2	2	AdvSM-3	2
		High	≥5			AdvSM-4	2–3
						AdvSM-5	4–5

Abbreviations: MARS, Mutation-Adjusted Risk Score for advanced systemic mastocytosis; MAPS, Mayo Alliance Prognostic System for mastocytosis; IPSS, International Prognostic Scoring System for non-advanced systemic mastocytosis; S/A/R, presence of *SRSF2* and/or *ASXL1* and/or *RUNX1* mutation; *SRFS2*, Serine And Arginine Rich Splicing Factor 2; *ASXL1*, Additional Sex Combs-Like Transcriptional Regulator 1; *RUNX1*, Runt-related transcription factor 1 SM, systemic mastocytosis; ISM, indolent systemic mastocytosis; SSM, smoldering systemic mastocytosis; ALP, alkaline phosphatase; *NRAS*, NRAS proto-oncogene; AdvSM, advanced systemic mastocytosis.

**Table 5:** Principal recruiting clinical trials involving SM.

Clinicaltrials.Gov Identifier	Intervention	Title	Primary Outcome Measures
NCT03770273	Sarilimus	A Phase 2 Randomized Double-Blinded Placebo-Controlled Study to Evaluate the Safety and Efficacy of Subcutaneous Sarilumab in Improving the Quality of Life in Subjects with Indolent Systemic Mastocytosis	Frequency and severity of adverse events (AEs); mastocytosis Quality of Life Questionnaire (MC-QoL)
NCT04333108	Masitinib	Phase 3 Study to Compare Oral Masitinib to Placebo in Treatment of Patients with Smouldering or Indolent Severe Systemic Mastocytosis, Unresponsive to Optimal Symptomatic Treatment	Cumulative response in at least one of three severe baseline symptoms of mast cell mediator release (pruritus, flushes, or depression).
NCT04910685	BLU-263	A Randomized, Double-Blind, Placebo-Controlled Phase 2/3 Study of BLU-263 in Indolent Systemic Mastocytosis	Recommended Dose (RD) in patients with ISM; response rate in patients with ISM; long-term safety and tolerability of BLU-263 as assessed by the number of adverse events and serious adverse events; mean change in Indolent Systemic Mastocytosis, Symptom Assessment Form (ISM-SAF) Total Symptom Score (TSS)
NCT03731260	Avapritinib (BLU-285)	A 3-Part, Randomized, Double-Blind, Placebo-Controlled Phase 2 Study to Evaluate Safety and Efficacy of Avapritinib (BLU-285), a Selective *KIT* Mutation-Targeted Tyrosine Kinase Inhibitor, in Indolent and Smoldering Systemic Mastocytosis With Symptoms Inadequately Controlled With Standard Therapy	Recommended Phase 2 dose (RP2D) in patients with ISM; proportion of responders, defined as ≥30% reduction in ISM Symptom Assessment Form
NCT04996875	Bezuclastinib (CGT9486; PLX9486)	A Phase 2 Open-Label, Multicenter Clinical Study of the Safety, Efficacy, Pharmacokinetic, and Pharmacodynamic Profiles of CGT9486 as a Single Agent in Patients With Advanced Systemic Mastocytosis	Determine the optimal dose of CGT9486 by safety assessments and response criteria; objective response rate according to modified IWG-MRT-ECNM response criteria
NCT03214666	GTB-3550	GTB-3550 (CD16/IL-15/CD33) Tri-Specific Killer Engager (TriKE™) for the Treatment of HighRisk Myelodysplastic Syndromes, Refractory/Relapsed Acute Myeloid Leukemia and Advanced Systemic Mastocytosis	Maximum Tolerated Dose (MTD) of GTB-3550 TriKE™ finding; incidence of complete and partial remission due to GTB-3550 TriKE™ treatment
NCT04681105	Flotetuzumab	A Phase 1 Trial to Evaluate the Safety of Single Agent Flotetuzumab in Advanced CD123-Positive Hematological Malignancies	Maximum tolerated dose (recommended phase 2 dose, RP2D) of flotetuzumab; evaluate the safety and tolerability of flotetuzumab in CD123-positive advanced ALL) (Cohort A) and other hematological malignancies (Cohort B), by evaluation of toxicities including: type, frequency, severity, attribution, and duration of the toxicity.

## Data Availability

Not applicable.
